# Biochemical shifts in *Chlorella vulgaris* via post-stationary magnesium sulfate stress: optimizing biomass for advanced bio-fertilizers

**DOI:** 10.3389/fpls.2026.1867866

**Published:** 2026-06-09

**Authors:** Charith Akalanka Dodangodage, Jagath C. Kasturiarachchi, Thilini A. Perera, Sanjitha Dilan Rajapakshe, Sayuri S Niyangoda, Rangika Umesh Halwatura

**Affiliations:** 1 ProGreen Lab, Department of Civil Engineering, University of Moratuwa, Moratuwa, Sri Lanka; 2 Department of Applied Sciences, Sri Lanka Institute of Information Technology, Malabe, Sri Lanka; 3 Department of Plant Sciences, University of Colombo, Colombo, Sri Lanka; 4 Department of Biosystems Technology, Uva Wellassa University, Badulla, Sri Lanka; 5 Department of Chemistry, University of Kansas, Lawrence, KS, United States

**Keywords:** biphasic cultivation, carbohydrate enrichment, *Chlorella vulgaris*, lipid accumulation, microalgal bio-fertilizer, osmotic stress

## Abstract

Sustainable agriculture requires bio-fertilizers that improve both nutrient efficiency and soil resilience. Microalgae are promising candidates; however, conventional optimization using sodium chloride (NaCl) stress introduces phytotoxic sodium residues that limit soil application. To address this, a biphasic cultivation strategy for *Chlorella vulgaris* was developed using magnesium sulfate (MgSO_4_) as a dual-function stressor. Following the onset of a nitrogen-limited stationary phase (Day 18), the addition of 0.4 g L^-^¹ MgSO_4_ induced intracellular macromolecular accumulation. Biomass increased by 44.8% (2.810 ± 0.090 g L^-^¹), driven by intracellular densification, with enrichment in both total carbohydrate (42.15 ± 2.10%) and lipid (36.24 ± 1.11%) fractions. Substituting NaCl with MgSO_4_ eliminates the risk of sodium-induced phytotoxicity upon soil application, while simultaneously pre-loading the biomass with essential secondary macronutrients. Overall, this study demonstrates that targeted MgSO_4_-induced metabolic shifts can generate high-density, functionally enhanced, sodium-free microalgal biomass to serve as a potential bio-fertilizer feedstock.

## 1 Introduction

Global agriculture must urgently increase crop productivity while mitigating the environmental burden of inefficient synthetic fertilizers, which often exhibit nutrient use efficiencies (NUE) below 50% (Brunelle et al., 2024; Liu et al., 2025). The unabsorbed remainder is lost via leaching and surface runoff, driving environmental crises such as groundwater eutrophication and greenhouse-gas emissions (Awadelkareem et al., 2023; Lal, 2015). Furthermore, conventional fertilizers frequently under-supply essential secondary macronutrients like magnesium (Mg) and sulfur (S) (Z. Wang et al., 2020). This chronic deficit accelerates soil acidification and limits crop yields by approximately 8–11%, whereas optimized Mg and S management supports long-term structural fertility and stress tolerance (Agegnehu et al., 2021; Chaudhry et al., 2021; Dick et al., 2008).

To address these sustainability challenges, agricultural research has increasingly explored microalgae as potential bio-fertilizer feedstocks. The valorization of waste streams into functional materials is a critical component of the circular economy, spanning applications from composites to microalgal biomass (Fernando et al., 2026; Dodangodage et al., 2026a). Unlike conventional organic amendments, microalgae offer a tunable biochemical composition, nutrient recovery capabilities, and photosynthetic carbon capture (Johnson et al., 2025; Song et al., 2024). These systems can actively enhance soil fertility, nutrient cycling, and carbon-nitrogen dynamics (Sun et al., 2026). *Chlorella vulgaris* serves as a practical bioengineering platform due to its rapid autotrophic growth and inherent capacity to synthesize complex carbohydrates—including extracellular polymeric substances (EPS)—and intracellular lipids under controlled conditions (Piasecka and Baier, 2022; Dodangodage et al., 2026a).

To optimize microalgal biomass for agronomic use, controlled abiotic stress is frequently deployed to redirect intracellular carbon away from growth-associated protein synthesis and toward storage compounds like carbohydrates and lipids (Gupta et al., 2025; Wu et al., 2024). While sodium chloride (NaCl) effectively induces osmotic stress, it introduces toxic Na^+^ residues that induce secondary soil salinization and trigger phytotoxicity, rendering the resulting biomass detrimental for soil application (Vangenechten et al., 2025; Zhao et al., 2019). Although a direct experimental comparison with NaCl-treated cultures was not included in this study, the rationale for substituting NaCl is firmly grounded in this extensively documented sodium-induced phytotoxicity. Consequently, magnesium sulfate (MgSO_4_) emerges as an alternative dual-function stressor. It provides the necessary osmotic pressure to drive targeted metabolic shifts without sodium toxicity, while simultaneously supplying essential macronutrients (Mg²^+^ and SO_4_²^-^) that are assimilated into intracellular components (Paliwal et al., 2017; Suparmaniam et al., 2024). By utilizing MgSO_4_, we hypothesize that carbon-flux stress can be induced to generate a nutrient-dense biomass with potential for agricultural application.

The effectiveness of these metabolic shifts depends heavily on the timing of stress application. Applying stressors during the exponential growth phase abruptly halts cell division, reducing overall biomass yields (Shi et al., 2020; J. Wang et al., 2022). In contrast, imposing stress at the onset of the stationary phase creates a biphasic cultivation pathway. Because cellular proliferation has plateaued, metabolism is redirected toward lipid and carbohydrate synthesis without sacrificing the accumulated baseline biomass (Chiranjeevi and Venkata Mohan, 2016). However, a critical research gap persists. Systematic evaluation of MgSO_4_-induced modulation under post-stationary phase conditions remains limited, and contemporary literature rarely explores how manipulating these intracellular responses can optimize the biochemical composition of the final biomass for soil application (Guieysse and Plouviez, 2024; Novoveská et al., 2023).

Therefore, the central hypothesis of this study is that applying MgSO_4_ as an osmotic stressor during the post-stationary phase will trigger metabolic carbon flux toward carbohydrate and lipid biosynthesis in *Chlorella vulgaris*, avoiding the phytotoxic sodium residues associated with traditional NaCl stress. To test this hypothesis and optimize the biomass as a potential bio-fertilizer feedstock, the specific objectives of this study were to: (1) evaluate the effect of a post-stationary MgSO_4_ gradient (0.1 to 0.5 g L^-^¹) on the biphasic growth kinetics and biomass yield of *Chlorella vulgaris*; (2) quantify the subsequent metabolic shifts in intracellular macromolecular composition, specifically the enrichment of carbohydrates and lipids; and (3) assess the biochemical suitability of the resulting biomass as a potential sodium-free, nutrient-dense agricultural amendment.

## 2 Materials and methods

### 2.1 Microalgal strain and inoculum preparation

A pure culture of *Chlorella vulgaris* (Strain ID: PG-CHL-01) was obtained from the ProGreen Laboratory, University of Moratuwa, Sri Lanka. Stock cultures and pre-cultures were maintained in sterilized Bold’s Basal Medium (BBM) under autotrophic conditions at 25 ± 2 °C, with continuous (24 h) illumination at 150 µmol photons m^-^² s^-^¹ provided by cool-white LED lamps and continuous aeration at 0.5 vvm using sterile-filtered ambient air (~0.04% CO_2_). Exponentially growing pre-cultures were used as inocula in all experiments to minimize lag-phase effects after transfer and to ensure a physiologically active and uniform starting population. Prior to inoculation, the biomass concentration of the inoculum was standardized to 0.03 g L^-^¹ using a gravimetric dry-weight calibration curve established between optical density and dry cell weight (Dodangodage et al., 2026a; Porninta et al., 2023; Mattos et al., 2012; Nicodemou et al., 2024).

### 2.2 Culture medium composition and salinity preparation

All experiments were conducted using standard Bold’s Basal Medium (BBM) prepared with distilled water according to the conventional formulation, dispensed into suitable vessels, and sterilized by autoclaving for 20 min. Osmotic stress was imposed by supplementing the sterile basal medium with analytical-grade magnesium sulfate heptahydrate (MgSO_4_·7H_2_O) as a dual-function stressor. A concentrated MgSO_4_·7H_2_O stock solution was prepared separately in distilled water, sterilized independently, and aseptically added to the cooled sterile BBM to achieve the required final salinity levels.

For the screening stage, MgSO_4_·7H_2_O was applied across a gradient of 0.1, 0.2, 0.3, 0.4, and 0.5 g L^-^¹, while unsupplemented BBM containing 0 g L^-^¹ served as the control. Prior to inoculation, the initial pH of all media was adjusted using sterile 0.1 M HCl or 0.1 M NaOH as required to ensure that the stressor concentration remained the sole independent variable at the start of cultivation (Dodangodage et al., 2026b). To quantitatively characterize the applied osmotic stress, the thermodynamic parameters of the optimized 0.4 g L^-1^ MgSO_4_·7H_2_O treatment were calculated. This concentration corresponds to a theoretical molarity of 1.62 mM, contributing an osmolarity of 3.24 mOsm L^-1^ and an ionic strength of 6.48 mM to the basal medium.

### 2.3 Experimental design and biphasic cultivation strategy

The study was conducted as a two-stage experimental investigation to evaluate the effects of MgSO_4_-induced osmotic stress on the biochemical characteristics of *Chlorella vulgaris*. All experimental conditions were conducted in triplicate independent reactors.

#### 2.3.1 Stage I (Salinity screening)

The screening experiment was conducted using BBM supplemented with MgSO_4_·7H_2_O at the specified gradient. Cultivation was carried out in culture vessels with a working volume of 400 mL in 500 mL containers under identical environmental and operating conditions. The objective of this stage was to identify a stress level that maximized macromolecular accumulation under mild osmotic stress without inducing cellular toxicity.

#### 2.3.2 Stage II (Main cultivation experiment)

Based on the results of the screening stage, the optimal MgSO_4_·7H_2_O treatment and the control were carried forward. Batch cultivation was performed in 2 L borosilicate glass bottles fitted with GL45 screw caps, with a working volume of 1.8 L per vessel. Cultures were maintained at 25 ± 2 °C under continuous (24 h) illumination at 150 µmol photons m^-^² s^-^¹ and continuously aerated at 0.5 vvm with ambient air (~0.04% CO_2_). The stressor was explicitly introduced at the onset of the stationary phase to isolate biomass proliferation from stress-induced metabolite synthesis. The onset of the stationary phase was quantitatively identified based on the stabilization of optical density, strictly defined as a variance of less than 5% in OD_680_ over a 48-h monitoring period (Dodangodage et al., 2026a, 2026).

### 2.4 Growth monitoring and kinetic calculations

#### 2.4.1 Optical density

Biomass growth was monitored periodically by measuring the optical density of the cultures at 680 nm (OD_680_) using a UV-Vis spectrophotometer (Shimadzu UV-1800, Kyoto, Japan). Prior to measurement, each culture was gently mixed. When the measured absorbance exceeded the linear range of the instrument, samples were appropriately diluted with the corresponding blank medium and corrected using the relevant dilution factor. A calibration curve correlating OD_680_ to DCW was established and used for rapid biomass estimation (Dodangodage et al., 2026d). The established linear regression equation was DCW = [0.81] x OD_680_ + [0.12] (R^2^ = 0.98), confirming a highly proportional relationship within the measured absorbance range.

#### 2.4.2 Dry cell weight

Dry cell weight (DCW) was determined gravimetrically to quantify biomass concentration. A 10 mL aliquot of well-mixed culture was filtered through pre-dried and pre-weighed glass microfiber filters (Hyundai GF/C, 1.2 µm pore size). The loaded filters were dried at 60 °C until constant weight, cooled in a desiccator, and reweighed. Biomass concentration was calculated according (Dodangodage et al., 2026d) to Equation 1:

(1) $$DCW_{n}=\frac{W_{f}-W_{i}}{V}$$

where $DCW_{n}$ is the biomass concentration at sampling day n (g L-^1^), $W_{f}$ is the final mass of the dried filter plus retained biomass (g), $W_{i}$ is the initial mass of the dried filter (g), and V is the filtered sample volume (L).

#### 2.4.3 Growth kinetics and biomass productivity

The specific growth rate μ during the exponential growth phase, identified from the linear region of ln(DCW) versus time, was calculated according (Trenkenshu, 2019) to Equation 2:

(2) $$\mu =\frac{\text{ln}(X_{2}/X_{1})}{t_{2}-t_{1}}$$

where $X_{1}$ and $X_{2}$ are the biomass concentrations (g L^-^¹) at times $t_{1}$ and $t_{2}$, respectively. Biomass productivity $P_{\text{biomass}}$ was calculated according to Equation 3:

(3) $$P_{\text{biomass}}=\frac{X_{f}-X_{i}}{t}$$

where $P_{\text{biomass}}$ is the biomass productivity (g L^-1^ d^-1^), $X_{i}$ is the initial biomass concentration (g L^-1^), $X_{f}$ is the final biomass concentration (g L^-1^), and t is the cultivation time (d).

### 2.5 Culture chemistry and nutrient profiling

To distinguish salinity-induced responses from changes associated with medium depletion, culture pH and dissolved nutrient concentrations were monitored at two-day intervals throughout cultivation. All analyses were performed using the clarified supernatant obtained after the removal of suspended biomass by centrifugation at 4000 rpm for 20 min and subsequent filtration through a 0.22 µm syringe filter.

#### 2.5.1 pH Monitoring

Culture pH was measured using a calibrated benchtop pH meter (SevenCompact, Mettler Toledo, Switzerland). Measurements were carried out immediately after sample collection to minimize changes associated with atmospheric CO_2_ exchange.

#### 2.5.2 Nitrate analysis

Nitrate concentration was determined spectrophotometrically using the ultraviolet absorbance method at 220 nm with baseline correction at 275 nm. Absorbance was measured against an appropriate reagent blank, and nitrate concentration was quantified using a calibration curve prepared from analytical-grade potassium nitrate (KNO_3_) standards (Dodangodage et al., 2026d; Fitri et al., 2020).

#### 2.5.3 Phosphate analysis

Phosphate concentration was determined by the molybdenum blue spectrophotometric method at 880 nm. Quantification was performed using a calibration curve prepared from potassium dihydrogen phosphate (KH_2_PO_4_) standards using freshly prepared reagents (Dodangodage et al., 2026c) (Fitri et al., 2020).

#### 2.5.4 Nutrient removal efficiency

The removal efficiency of nitrate and phosphate during cultivation was calculated (Rita and Bragança, 2021) using Equation 4:

(4) $$RE(\%)=\frac{C_{i}-C_{t}}{C_{i}}\times 100$$

where RE is the nutrient removal efficiency (%), $C_{i}$ is the initial nutrient concentration (mg L^-1^), and $C_{t}$ is the nutrient concentration (mg L^-1^) at time *t*.

### 2.6 Biomass harvesting and analytical quantification

#### 2.6.1 Harvesting

Culture samples from each treatment were collected and concentrated by centrifugation at 4000 rpm for 20 min using a bench-top centrifuge (Eppendorf 5810R, Hamburg, Germany). The recovered biomass pellets were washed twice with distilled water, dried at 60 °C until constant weight was achieved, gently ground to obtain a homogeneous sample, and stored in a desiccator until subsequent biochemical analysis (Potts, 2000; Dodangodage et al., 2026a).

#### 2.6.2 Total carbohydrate content quantification

Total cellular carbohydrate content was determined using a modified phenol-sulfuric acid method following pigment removal and acid hydrolysis. Dried biomass (10 mg) was treated with 0.5 mL of acetic acid and heated at 80 °C for 20 min. Pigments were removed by adding 10 mL of acetone followed by centrifugation at 3500 x g for 10 min. The resulting pellet, containing the bulk cellular biomass -was retained, ensuring no target carbohydrates were discarded during the solvent wash. The pellet was then hydrolyzed with 2.5 mL of 4 M trifluoroacetic acid (TFA) at 95 °C for 4 h. The total carbohydrate content of the supernatant was quantified spectrophotometrically using glucose as the calibration standard and reported as percentage dry weight (%DW) (Jia et al., 2015; Dubois et al., 1956).

#### 2.6.3 Lipid content quantification

Total lipid content was determined using a modified Bligh and Dyer solvent extraction method (Bligh and Dyer, 1959). To accommodate the mass balance limitations of the 1.8 L cultivation volume across multiple sampling time points, the standard extraction protocol was proportionally scaled down. Dried biomass (50 mg) was homogenized in a chloroform-methanol solvent system (2:1, v/v). Phase separation was induced by the addition of saline solution, allowing the partitioning of lipids into the lower organic phase. The chloroform layer was evaporated to constant weight, and total lipid content was determined gravimetrically and expressed as %DW. All extractions were performed in triplicate to ensure analytical reproducibility (Lee et al., 2013; Akondi et al., 2017).

### 2.7 Statistical analysis

All experiments were conducted in independent triplicates (n = 3), and the results are presented as mean ± standard deviation (SD). Prior to conducting inferential statistics, the normality of the data distribution and the homogeneity of variances were explicitly verified using the Shapiro-Wilk test and Levene’s test, respectively. Differences among treatments in the screening stage were evaluated using one-way analysis of variance (ANOVA), followed by Tukey’s *post hoc* multiple comparison test. Comparisons between the selected treatment and the control were performed using the same significance threshold. Statistical analyses were carried out using Minitab 17 (Minitab Inc., USA), and graphical figures were prepared using Microsoft Excel (Office 365). Statistical significance was defined as p < 0.05, and distinct superscript letters (e.g., a, b, c) are utilized to denote statistically significant differences between specific treatment groups.

## 3 Results

### 3.1 Screening of salinity conditions and selection of the optimal MgSO_4_ level

**Figure 1:**
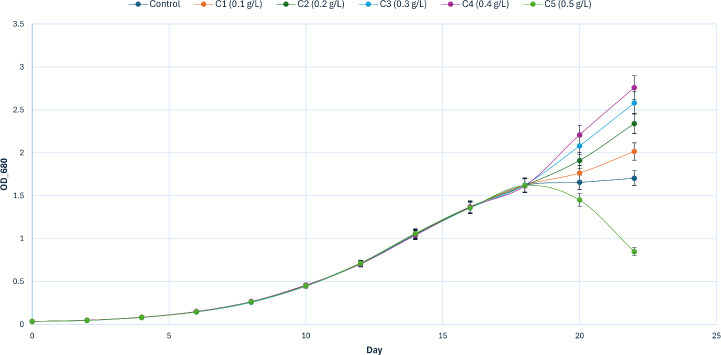
Dose-dependent divergence of post-stress growth trajectories following the addition of varying MgSO_4_ concentrations (0.1 to 0.5 g L^-^¹).

The preliminary screening experiment was conducted to identify the MgSO_4_ concentration that maximized biomass accumulation in *Chlorella vulgaris* without inducing cellular toxicity under the proposed biphasic cultivation strategy. Growth kinetics were monitored periodically via optical density (OD_680_) (**Figure 1**) and dry cell weight (DCW) to establish a stable baseline prior to stress induction.

During the pre-stress exponential and early stationary phases (Days 0–18), all cultures exhibited identical growth trajectories under uniform basal conditions. By Day 18, the OD_680_ for all experimental vessels converged at approximately 1.62, while the corresponding DCW reached an average of 1.35 ± 0.04 g L^-^¹. This confirms that all cultures entered the stress phase from statistically equivalent physiological states.

**Figure 2:**
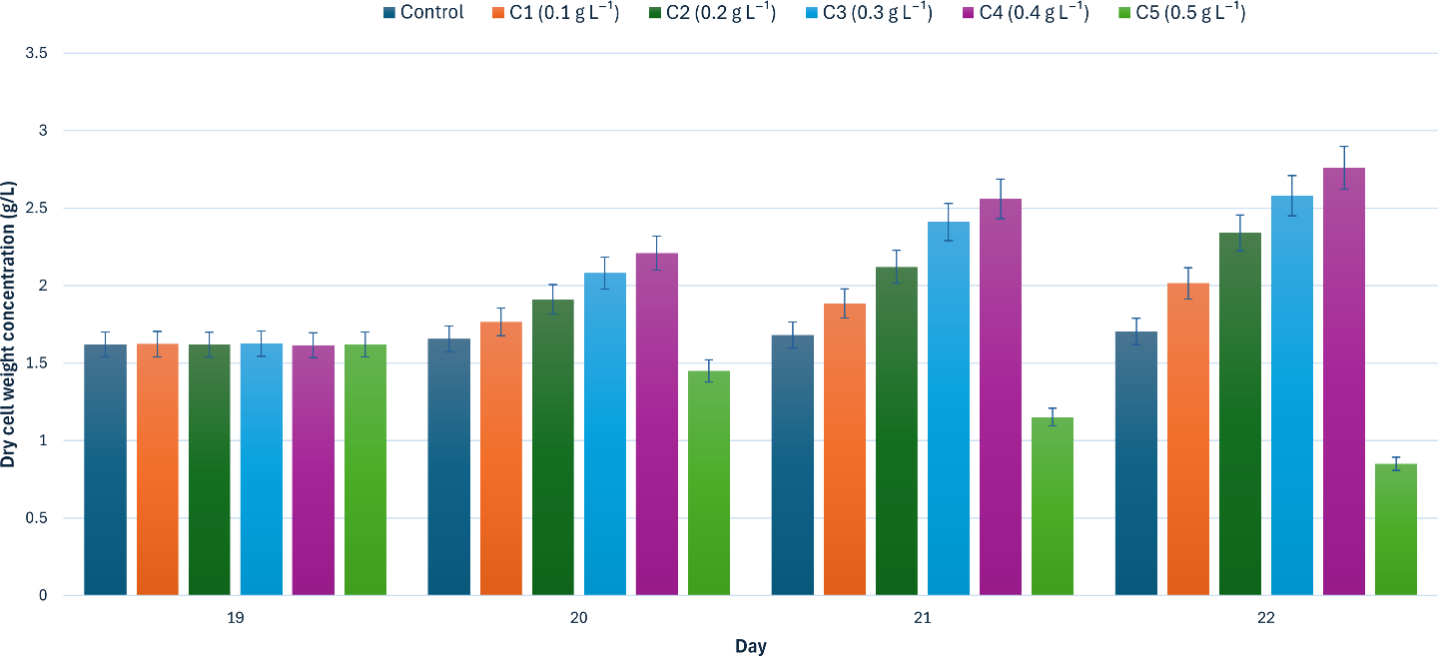
Dry cell weight (DCW) progression of Chlorella vulgaris. establishing a physiological baseline to stress induction (Days19 - 22).

Following the aseptic introduction of the MgSO_4_ stressor on Day 19 (starting from a corresponding DCW of approximately 1.38 ± 0.04 g L^-^¹), the growth trajectories diverged significantly in a dose-dependent manner (**Figure 2**). The unstressed control culture exhibited minimal post-stationary growth, stabilizing at a final OD_680_ of 1.704 ± 0.048, corresponding to a DCW of 1.70 ± 0.05 g L^-^¹ by Day 22. In contrast, cultures treated with moderate MgSO_4_ concentrations (0.1 to 0.4 g L^-^¹) demonstrated a distinct secondary phase of mass accumulation. This post-stress increment scaled positively with the applied osmotic gradient up to a critical threshold, indicating that the induced stress sustained biomass accumulation beyond the natural stationary phase plateau.

A quantitative comparison of the final biomass yields after 72 hours of stress exposure (Day 22) revealed statistically significant differences among the treatments (based on ANOVA followed by Tukey’s *post hoc* test, p < 0.05) (**Table 1**). The maximum final biomass was achieved in the 0.4 g L^-^¹ MgSO_4_ treatment (C4), which reached a peak DCW of 2.760 ± 0.095 g L^-^¹. This represented a substantial 61.9% increase relative to the final control biomass and was statistically superior to the 0.1, 0.2, and 0.3 g L^-^¹ treatments.

**Table 1:** Final biomass yields (DCW) and statistical significance across varying MgSO_4_ concentrations after 72 hours of stress exposure (Day 22).

Treatment	Before salt addition (Day 19)	After 1 Day (Day 20)	After 2 Days (Day 21)	After 3 Days (Day 22)
Control	1.620 ± 0.036	1.656 ± 0.048	1.680 ± 0.060	1.704 ± 0.048^c^
C1 (0.1 g L^-^¹)	1.622 ± 0.040	1.765 ± 0.052	1.885 ± 0.060	2.015 ± 0.065^b^
C2 (0.2 g L^-^¹)	1.618 ± 0.038	1.910 ± 0.060	2.120 ± 0.075	2.340 ± 0.080^b^
C3 (0.3 g L^-^¹)	1.625 ± 0.042	2.080 ± 0.070	2.410 ± 0.085	2.580 ± 0.090^a^
C4 (0.4 g L^-^¹)	1.615 ± 0.035	2.210 ± 0.075	2.560 ± 0.090	2.760 ± 0.095^a^
C5 (0.5 g L^-^¹)	1.620 ± 0.044	1.450 ± 0.055	1.150 ± 0.065	0.850 ± 0.050^d^

Values represent mean ± SD (n = 3). Different superscript letters (a, b, c, d) within the Day 22 column indicate statistically significant differences between treatments (p < 0.05).

However, further elevating the salinity stress to 0.5 g L^-^¹ (C5) proved inhibitory. At this highest concentration, the culture experienced a decline, with the DCW decreasing from 1.38 ± 0.04 g L^-^¹ on Day 19 to 0.850 ± 0.050 g L^-^¹ by Day 22. This decline indicates that the 0.5 g L^-1^ concentration exceeded the cellular osmotic tolerance threshold, likely inducing severe plasmolysis and subsequent cell lysis rather than metabolic acclimation. Consequently, the 0.4 g L^-^¹ MgSO_4_ concentration was identified as the optimal stress threshold capable of maximizing biomass accumulation without breaching the limits of cellular viability. This concentration was selected as the sole stress condition for all subsequent scaled-up cultivations and biochemical profiling.

### 3.2 Growth performance under the selected osmotic stress condition

#### 3.2.1 Growth profiles

**Figure 3:**
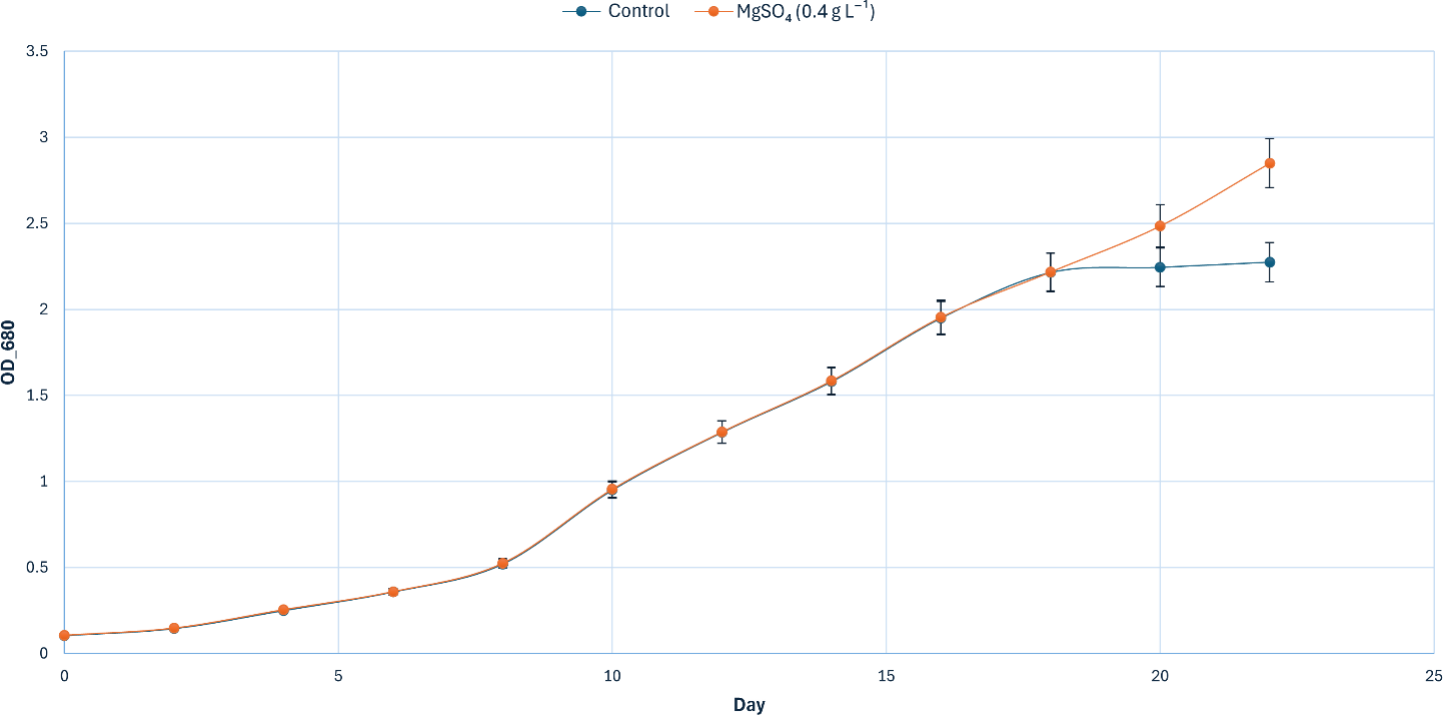
Optical density (OD_680_) profiles comparing the optimized 0.4 g L^-^¹ MgSO_4_ biphasic stress strategy against an unstressed control in a scaled-up 1.8 L cultivation system.

**Figure 4:**
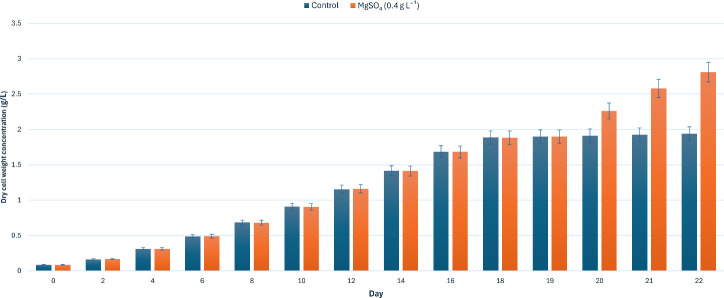
Dry cell weight (DCW) profiles demonstrating secondary mass accumulation under the optimized 0.4 g L^-^¹ MgSO_4_ stressor versus the unstressed control.

To validate the scalability and consistency of the biomass response identified during the initial screening, the optimized salinity condition of 0.4 g L^-^¹ MgSO_4_ was further evaluated against an unstressed control in a scaled-up 1.8 L batch cultivation system. Growth was monitored periodically via OD_680_ (**Figure 3**) and DCW (**Figure 4**) to provide a highly resolved comparison of the pre-stress and post-stress kinetics.

During the pre-stress autotrophic growth phase (Days 0–18), the control and the designated stress culture exhibited comparable growth kinetics. Both cultures demonstrated exponential proliferation, followed by a gradual deceleration as they approached the stationary phase. By Day 18, the OD_680_ values were 2.215 ± 0.050 for the control and 2.218 ± 0.048 for the target culture. This optical consistency was confirmed by the DCW measurements, which reached 1.885 ± 0.045 g L^-^¹ and 1.882 ± 0.042 g L^-^¹, respectively, confirming that both cultures entered the stress phase under equivalent physiological conditions.

Following the aseptic introduction of 0.4 g L^-^¹ MgSO_4_ on Day 19, the kinetic profiles of the two cultures diverged. The unstressed control culture exhibited only nominal mass accumulation throughout the remainder of the cultivation, characteristic of a standard stationary phase. By the peak harvest on Day 22, the control stabilized at a final OD_680_ of 2.275 ± 0.045 and a DCW of 1.940 ± 0.045 g L^-^¹.

Conversely, the culture subjected to the targeted MgSO_4_ stress exhibited a steady secondary accumulation phase without a corresponding increase in cell division, suggesting a potential shift toward intracellular carbon reallocation rather than proliferation. Within 24 hours post-stress (Day 20), the DCW of the treated culture increased to 2.260 ± 0.065 g L^-^¹, diverging from the control. This upward trajectory continued steadily through Day 21 (2.580 ± 0.080 g L^-^¹) and culminated at a final peak DCW of 2.810 ± 0.090 g L^-^¹ on Day 22. The corresponding OD_680_ similarly peaked at 2.850 ± 0.095.

Quantitative analysis of the final harvest (Day 22) confirmed that the 0.4 g L^-^¹ MgSO_4_ treatment yielded a 44.8% increase in final dry biomass compared to the unstressed control (p < 0.05). Importantly, this mass accumulation was driven by intracellular densification rather than cellular proliferation. Based on the established calibration curve (DCW = 0.81 x OD_680_ + 0.12, an OD_680_ of 2.850 corresponds to a predicted baseline dry weight of 2.42 g L^-1^. The surplus actual mass (2.810 g L^-1^) demonstrates that the cells accumulated dense intracellular storage metabolites without a proportional increase in optical density or cell division.

#### 3.2.2 Post-stress biomass productivity

To quantitatively evaluate the kinetic impact of the applied osmotic stress, biomass productivity was analyzed across the distinct cultivation phases. During the pre-stress autotrophic phase (Days 0–18), both cultures exhibited statistically indistinguishable specific growth rates and overall biomass productivities (approximately 0.10 g L^-^¹ d^-^¹), reflecting a parallel progression through the exponential and deceleration phases.

However, kinetic divergence became evident during the critical 72-hour post-stress temporal window (Days 19–22). Following its natural transition into the stationary phase, the mass accumulation rate of the unstressed control culture effectively plateaued. Between Day 19 (1.900 ± 0.048 g L^-^¹) and Day 22 (1.940 ± 0.045 g L^-^¹), the control yielded a negligible post-stress productivity of only 0.013 g L^-^¹ d^-^¹.

By comparison, the culture subjected to 0.4 g L^-^¹ MgSO_4_ maintained a sustained biomass accumulation rate. The between Day 19 (1.898 ± 0.045 g L^-^¹) and Day 22 (2.810 ± 0.090 g L^-^¹), the post-stress biomass productivity of the treated culture increased to 0.304 g L^-^¹ d^-^¹. This corresponds to a 23.4-fold increase in the mass accumulation rate relative to the control over the same time interval. These results indicate that the application of the MgSO_4_ dual-function stressor at the onset of the stationary phase appears to mitigate the typical stress-induced cessation of productivity under the conditions tested, enabling a secondary phase of biomass accumulation. This kinetic enhancement provides quantitative support for the hypothesis that post-stationary phase MgSO_4_ stress effectively decouples biomass accumulation from conventional growth limitations.

### 3.3 Extracellular pH dynamics during cultivation

**Figure 5:**
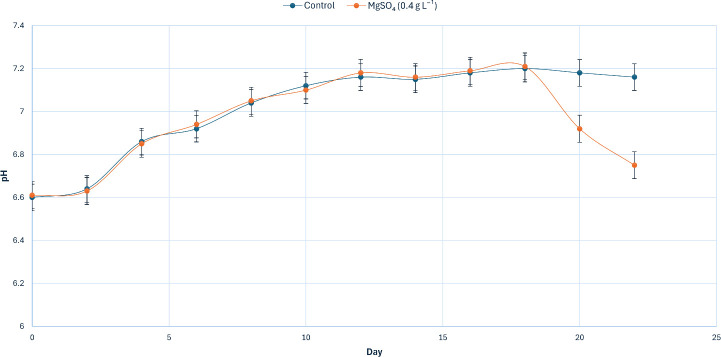
Extracellular pH dynamics during the pre-stress autotrophic growth and post-stress metabolic phases.

Changes in extracellular pH were monitored throughout the 1.8 L cultivation period to evaluate the macroscopic culture chemistry across the pre-stress and post-stress phases (**Figure 5**). At inoculation (Day 0), both the control and the 0.4 g L^-^¹ MgSO_4_-designated culture exhibited an identical baseline pH of approximately 6.60.

Throughout the active pre-stress autotrophic growth phase (Days 0–18), both cultures demonstrated a synchronized, gradual alkalization. This similar trajectory culminated on Day 18, reaching 7.20 ± 0.19 in the control and 7.21 ± 0.20 in the target culture, with no significant difference detected between the two systems (p > 0.05). This consistent alkalization is characteristic of active photosynthetic dissolved inorganic carbon assimilation and nutrient uptake.

Following the introduction of the MgSO_4_ dual-function stressor on Day 19, the pH profiles exhibited a treatment-dependent divergence. The unstressed control culture maintained a relatively stable extracellular pH, plateauing at 7.16 ± 0.15 by the final harvest on Day 22. This stabilization aligns with the previously established kinetic data (Section 3.2), reflecting a culture that has naturally settled into a steady stationary phase with balanced, low-level metabolic turnover.

Conversely, the culture subjected to osmotic stress experienced progressive acidification. Within 24 hours of MgSO_4_ addition (Day 20), the pH of the treated culture dropped to 6.92 ± 0.22, and declined further to a final value of 6.75 ± 0.18 by Day 22. Statistical analysis confirmed that the extracellular pH of the MgSO_4_-treated culture was significantly lower than that of the unstressed control during the entire post-stress period (p < 0.05). This statistically significant acidification is hypothesized to reflect active proton (H^+^) extrusion, an osmoregulatory response utilized to balance intracellular ion gradients. While this may indicate a metabolic shift, specific claims regarding enhanced organic acid production remain speculative, as no direct metabolite profiling was conducted in this study.

### 3.4 Nutrient utilization dynamics during cultivation

To determine whether the observed post-stress mass accumulation and pH shifts were driven by the applied osmotic stress rather than continued standard growth, macroscopic nutrient depletion profiles were periodically monitored.

#### 3.4.1 Nitrate depletion profile

**Figure 6:**
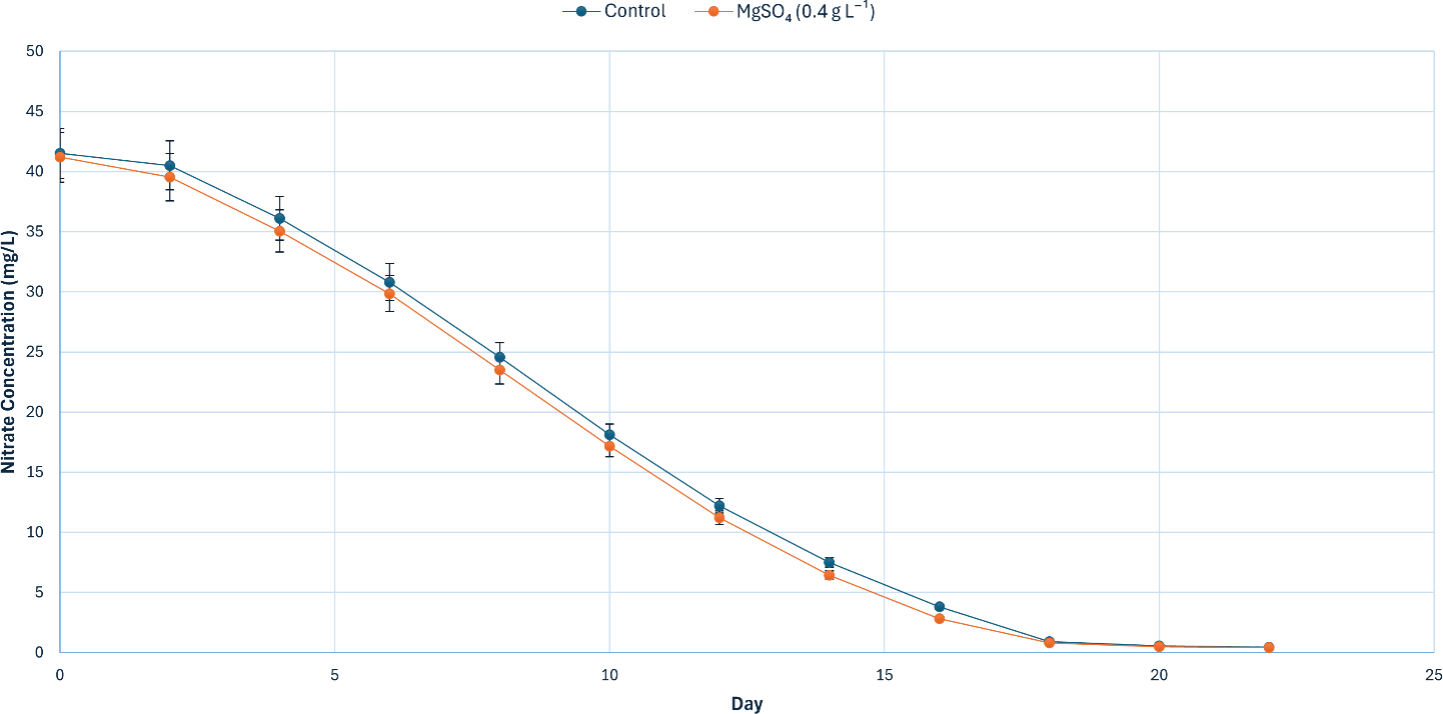
Nitrate-nitrogen (NO_3_^-^–N) depletion profiles confirming the near-complete exhaustion of nitrogen by Day 18 prior to stress induction.

Nitrate-nitrogen (NO_3_^-^–N) concentrations were tracked to define the exact onset of the nutrient-limited stationary phase (**Figure 6**). At inoculation (Day 0), the initial nitrate concentration in the culture medium was uniform across all reactors, averaging 41.15 ± 1.60 mg L^-^¹ for the control and 41.18 ± 1.55 mg L^-^¹ for the designated stress culture.

Throughout the active pre-stress autotrophic growth phase (Days 0–18), both cultures exhibited a rapid, parallel depletion of dissolved nitrate. This consumption trajectory closely mirrored the exponential biomass accumulation reported in Section 3.2. By Day 18, the nitrate concentration had plummeted to a near-exhausted state of 0.80 ± 0.05 mg L^-^¹ in the control and 0.82 ± 0.04 mg L^-^¹ in the target culture. Statistical analysis confirmed no significant difference in the nitrogen uptake rates or residual concentrations between the two systems prior to stress induction (p > 0.05).

Following the introduction of the 0.4 g L^-^¹ MgSO_4_ dual-function stressor on Day 19, the nitrate profiles fundamentally stabilized. Over the subsequent 72-hour stress period, nitrate levels remained negligible in both reactors, with the residual concentration slightly decreasing to a final plateau of 0.36 ± 0.02 mg L^-^¹ in the control and 0.42 ± 0.02 mg L^-^¹ in the treated culture by Day 22.

The near-complete exhaustion of nitrate coinciding with Day 18 is a critical process metric. These results collectively confirm effective nitrogen depletion by Day 18, establishing nitrogen limitation as the dominant constraint during the post-stress phase. Consequently, the observed post-stress physiological responses are more likely attributable to stress-induced metabolic shifts rather than continued nutrient-replete cellular proliferation.

#### 3.4.2 Phosphate depletion profile

**Figure 7:**
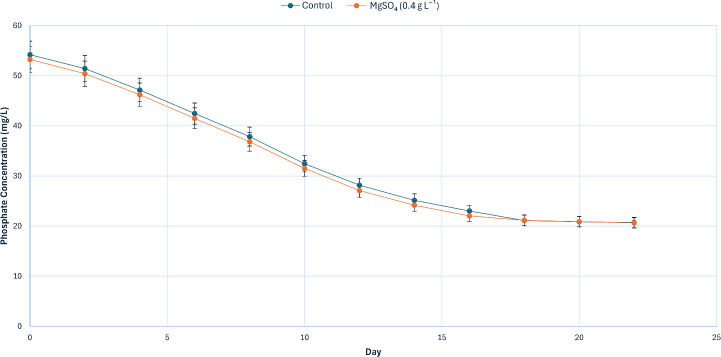
Extracellular phosphate-phosphorus (PO_4_³^-^–P) depletion profiles demonstrating residual phosphate availability during the nitrogen-limited stationary phase.

To complement the nitrogen utilization data and identify the primary limiting macronutrient, extracellular phosphate-phosphorus (PO_4_³^-^–P) depletion profiles were monitored concurrently (**Figure 7**). At the onset of cultivation (Day 0), the initial phosphate concentrations were statistically identical, recorded at 53.20 ± 2.55 mg L^-^¹ in the control and 53.25 ± 2.50 mg L^-^¹ in the designated target culture.

During the pre-stress exponential growth phase (Days 0–18), phosphate concentrations exhibited a steady, progressive decline that closely mirrored the concomitant biomass accumulation. As with the nitrate profiles, the phosphate consumption trajectories of both cultures remained tightly aligned, showing no significant variance (p > 0.05). By Day 18, the phosphate concentration had decreased to 21.10 ± 0.90 mg L^-^¹ in the control and 21.15 ± 0.88 mg L^-^¹ in the target culture.

Specifically, unlike nitrate - which was depleted to near-zero levels by this timepoint, a substantial residual phosphate pool representing approximately 39.7% of the initial concentration remained readily available in the medium.

Following the transition into the stationary phase and the subsequent addition of the 0.4 g L^-^¹ MgSO_4_ stressor on Day 19, phosphate uptake effectively ceased. Over the 72-hour post-stress monitoring period (Days 20–22), the extracellular phosphate levels stabilized entirely, concluding at 20.70 ± 0.90 mg L^-^¹ in the control and 20.65 ± 0.95 mg L^-^¹ in the treated culture by Day 22.

A substantial residual phosphate pool remained in the medium. In contrast, nitrate was nearly depleted. These results shows promise for support nitrogen depletion as the primary physiological constraint preceding stress induction.

### 3.5 Biochemical composition shifts under osmotic stress

To quantify the extent of macromolecular accumulation induced by the dual-function stressor under nitrogen-limited conditions, the temporal evolution of the primary biochemical fractions was analyzed. The daily progression of biomass concentration, total carbohydrate content (including extracellular polymeric substances, EPS), and total lipid content from the pre-stress baseline to the peak harvest is detailed in **Table 2**.

**Table 2:** Temporal evolution of the primary biochemical composition (total carbohydrates, EPS, and lipids) before and after dual-function stress induction.

Time (Days)	Treatment	Biomass (DCW, g/L)	Carbohydrate Content (DCW %)	Lipid Content (DCW %)
18 (Pre-Stress)	Control	1.885 ± 0.045	30.15 ± 1.50	18.20 ± 0.85
	MgSO_4_ (0.4 g/L)	1.882 ± 0.042	30.18 ± 1.45	18.25 ± 0.90
19 (Salt Added)	Control	1.900 ± 0.048	31.05 ± 1.60	18.80 ± 0.95
	MgSO_4_ (0.4 g/L)	1.898 ± 0.045	31.10 ± 1.55	18.85 ± 0.88
20 (After 1d)	Control	1.910 ± 0.048	32.16 ± 2.56	19.50 ± 1.05
	MgSO_4_ (0.4 g/L)	2.260 ± 0.065	35.50 ± 2.20	24.50 ± 1.05
21 (After 2d)	Control	1.925 ± 0.047	33.25 ± 2.10	20.40 ± 1.15
	MgSO_4_ (0.4 g/L)	2.580 ± 0.080	39.20 ± 2.45	30.80 ± 1.25
22 (Peak Harvest)	Control	1.940 ± 0.045^b^	34.50 ± 2.25^b^	21.80 ± 1.25^b^
	MgSO_4_ (0.4 g/L)	2.810 ± 0.090^a^	42.15 ± 2.10^a^	36.24 ± 1.11^a^

Values represent mean ± SD (n = 3). Different superscript letters (a, b) for the Day 22 (Peak Harvest) values indicate statistically significant differences between the Control and the MgSO_4_ treatment for that specific parameter (p < 0.05).

#### 3.5.1 Carbohydrate accumulation

Prior to the addition of the osmotic stressor, the carbohydrate contents of the control and the designated target culture were comparable, reflecting a consistent biochemical baseline. On Day 18 (pre-stress), the total carbohydrate fraction was 30.15 ± 1.50% for the control and 30.18 ± 1.45% for the MgSO_4_-designated culture. This parity was maintained through Day 19, immediately preceding stress induction (31.05 ± 1.60% and 31.10 ± 1.55%, respectively).

Following the aseptic introduction of 0.4 g L^-^¹ MgSO_4_ on Day 19, the biochemical composition began to diverge distinctly. The stressed culture exhibited a progressive accumulation of carbohydrates relative to control. By Day 20 (24 hours post-stress), the carbohydrate content in the treated biomass had accelerated to 35.50 ± 2.20%, whereas the unstressed control exhibited only a marginal increase to 32.16 ± 2.56%. This divergence widened significantly over the subsequent 48 hours, culminating in the carbohydrate fraction of the MgSO_4_-treated biomass reaching a maximum of 42.15 ± 2.10% by the peak harvest on Day 22. This constituted a statistically significant enrichment (p < 0.05) compared to the final control value of 34.50 ± 2.25%, indicating a significant increase in carbohydrate and EPS-associated fractions.

#### 3.5.2 Lipid accumulation

The lipid partitioning dynamics mirrored the rigorous baseline uniformity observed in the carbohydrate fractions. On Day 18, the intracellular lipid pools were indistinguishable, recorded at 18.20 ± 0.85% in the control and 18.25 ± 0.90% in the target culture. Both systems entered the stress phase on Day 19 with statistically equivalent lipid fractions of approximately 18.8%.

Upon exposure to the optimized 0.4 g L^-^¹ MgSO_4_ stressor, the targeted culture demonstrated a notable secondary enrichment in lipid content. Within 24 hours (Day 20), the lipid content of the treated culture surged to 24.50 ± 1.05%, distinctly separating from the control (19.50 ± 1.05%). The accumulation kinetics accelerated further through Day 21 (30.80 ± 1.25%). At the final Day 22 harvest, the MgSO_4_-treated biomass achieved an elevated final lipid content of 36.24 ± 1.11%. In contrast, the unstressed control, relying solely on natural stationary-phase lipid accumulation, reached only 21.80 ± 1.25%. Statistical evaluation confirmed that the 0.4 g L^-^¹ MgSO_4_ treatment induced a considerable increase (p < 0.05) in the lipid fraction, effectively nearly doubling the baseline lipid concentration within a 72-hour stress window.

## 4 Discussion

### 4.1 The trigger: nitrogen depletion as a prerequisite for metabolic shifts

The effectiveness of the biphasic cultivation strategy relies on the temporal separation of vegetative biomass proliferation and targeted macromolecular accumulation. Nutrient profiling (Section 3.4.1) confirmed the near-complete exhaustion of extracellular nitrate by Day 18. This nutrient limitation acted as the primary biological trigger, shifting the *Chlorella vulgaris* culture from an active exponential growth phase into a nutrient-limited stationary phase prior to stress induction.

As validated in two-stage microalgal systems, when extracellular nitrogen falls below a critical threshold, structural cell division is restricted, creating a physiological bottleneck where cells can no longer direct assimilated carbon toward vegetative growth (Henard et al., 2017; Yong et al., 2019; Sulochana and Arumugam, 2020). By deliberately introducing the targeted osmotic stressor strictly after the onset of nitrogen starvation (Day 19), it is hypothesized that the cells were restricted in synthesizing nitrogenous osmolytes. Instead, they were driven to upregulate the biosynthesis of carbon-rich, non-nitrogenous alternatives to manage the sudden MgSO_4_ osmotic gradient (Truong et al., 2024; Yaakob et al., 2021). This mechanistic sequence directly supports the rapid secondary phase of mass accumulation and the significant enrichment in both carbohydrate and lipid fractions observed within the 72-hour stress window.

### 4.2 Intracellular carbon flux and the activation of metabolic sinks

The significant accumulation of carbohydrates and lipids following the dual-function stress induction reflects a coordinated shift toward storage metabolite biosynthesis. Under conditions of nitrogen starvation, microalgae naturally downregulate the biosynthesis of nitrogen-dense macromolecules, yet the initial stages of the photosynthetic electron transport chain continue to function. This physiological decoupling is hypothesized to generate a continuous surplus of ATP and NADPH (Gupta et al., 2025; Lazrak et al., 2024).

To mitigate potential oxidative stress and rebalance the intracellular redox state caused by this theorized energy imbalance and the added MgSO_4_ osmotic shock, the cells likely activated alternative metabolic sinks (Zhu et al., 2018; Dodangodage et al., 2026d). The carbon assimilated from ongoing basal photosynthesis is redirected into the biosynthesis of nitrogen-free energy storage compounds (Li et al., 2021). Initially, this was observed as a statistically significant enrichment in the total carbohydrate fraction to 42.15 ± 2.10%, where secreted polysaccharides are proposed to serve both as an energy buffer and a structural osmoprotective barrier against the MgSO_4_-induced osmotic gradient (Esteves et al., 2025; Singh et al., 2024).

Concurrently, as the dual-stress environment persisted, lipogenesis was upregulated. The observed accumulation of lipids to 36.24 ± 1.11% is consistent with the hypothesis that lipogenesis acts as a major electron sink to safely sequester the persistent excess of ATP and NADPH under stress conditions (Narayanan et al., 2025; R. Wang and Miao, 2022). Therefore, the optimized 0.4 g L^-^¹ MgSO_4_ treatment successfully exploited the intrinsic photoprotective mechanisms of *Chlorella vulgaris*, driving targeted macromolecular accumulation.

### 4.3 The paradigm shift: MgSO_4_ vs. NaCl and the circular bioeconomy

While hyperosmotic stress is a well-documented trigger for secondary metabolite accumulation in microalgae - stimulating compounds that scavenge reactive oxygen species (ROS) and facilitate osmoregulation (Kolackova et al., 2023), the vast majority of contemporary literature relies on sodium chloride (NaCl) as the primary inducing agent. Although effective for biofuel-oriented lipogenesis, the use of NaCl presents a physiological constraint for potential bio-fertilizer feedstock applications. The application of NaCl-stressed biomass inherently introduces toxic sodium ions into the rhizosphere. Repeated field application of such biomass risks cumulative soil salinization, structural degradation (sodicity), and acute sodium toxicity in non-halophytic crops, primarily because Na^+^ actively disrupts K^+^ uptake and root osmotic balance (H. Yang and Hu, 2020). Furthermore, conventional NaCl-based systems generate hypersaline spent media, creating disposal challenges that undermine the sustainability of the fertilizer application (Fiorentino et al., 2025).

The selection of MgSO_4_ in this study strategically mitigates this limitation by functioning as a dual-action stressor. Mechanistically, the optimized 0.4 g L^-^¹ MgSO_4_ concentration likely provided a comparable hyperosmotic effect to conventional NaCl treatments, which is hypothesized to trigger the intracellular carbon rerouting toward osmolytes and lipids without introducing sodium toxicity (Kolackova et al., 2023). However, unlike NaCl, MgSO_4_ acts simultaneously as an agronomic pre-loading mechanism. Studies demonstrate that *Chlorella* species actively absorb Mg²^+^ (accumulating intracellularly to support key cellular functions, including the central coordination of the chlorophyll porphyrin ring), while the assimilated SO_4_²^-^ is indispensable for the biosynthesis of defense-related proteins and essential sulfur-containing amino acids, such as cysteine and methionine (Ayed et al., 2015). By utilizing MgSO_4_, the *Chlorella vulgaris* cells are induced to osmoregulate while concurrently sequestering these vital secondary macronutrients within their cellular matrix.

Furthermore, this chemical substitution represents a shift toward circular bioeconomy principles in microalgal bioprocessing, mirroring parallel strategies that valorize complex effluents into functional biochemicals (Dodangodage et al., 2026d). In conventional NaCl-based systems, both the harvested biomass and the residual cultivation medium become environmental challenges requiring costly downstream desalination (Fiorentino et al., 2025). In contrast, the MgSO_4_-driven strategy moves the system closer to a low-waste or circular process configuration. The stress-inducing agent is proposed to enhance the biostimulant potential of the final biomass, and any residual culture medium retains utilizable nutrients without the risk of toxicity. This dual functionality demonstrates upstream process engineering for sustainable nutrient cycling, ensuring that the bioprocess aligns well with closed-loop agricultural principles (J. Wang et al., 2022; Dodangodage et al., 2026b).

### 4.4 Limitations and future scale-up perspectives

While the 1.8 L laboratory-scale results demonstrate the efficacy of MgSO_4_-induced macromolecular accumulation, translating this biphasic strategy to commercial photobioreactors or raceway ponds introduces inherent scale-up challenges. Specifically, the dense accumulation of biomass (2.81 g L^-^¹) and the high carbohydrate fraction (42.15%) will alter culture rheology, potentially restricting gas-liquid mass transfer and light penetration at industrial volumes (Angelaalincy et al., 2017; Tigerprints and Walker, 2023).

Furthermore, it is necessary to acknowledge the analytical constraints of this study. The quantification of metabolic shifts relied on bulk biochemical assays (total carbohydrates and lipids) and macroscopic extracellular pH monitoring. To definitively validate the hypothesized intracellular redox imbalances (ATP/NADPH) and confirm the specific organic acids mediating proton extrusion, future research must incorporate advanced metabolomic profiling and transcriptomic analysis.

Finally, while the structural recalcitrance of the lipid-enriched biomass suggests sustained-release potential, actual field-scale mineralization kinetics and moisture retention must be empirically validated within a dynamic soil microbiome (G. Yang et al., 2025; Sarhan et al., 2024). Therefore, future research must prioritize advanced reactor engineering to maintain bioprocess efficiency at scale, coupled with *in vivo* soil column incubations and greenhouse trials to definitively quantify the real-world agronomic performance of this agricultural amendment candidate.

## 5 Conclusion

This study demonstrates that an optimized 0.4 g L^-^¹ MgSO_4_ treatment, applied following nitrogen depletion, functions as an effective dual-purpose osmotic stressor in *Chlorella vulgaris*, driving biomass densification and targeted macromolecular accumulation. The biphasic strategy achieved a 44.8% increase in final dry biomass, alongside an enrichment in both total carbohydrate (42.15%) and lipid (36.24%) fractions. Substituting traditional NaCl with MgSO_4_ eliminates the risk of sodium-induced phytotoxicity upon soil application, while simultaneously pre-loading the biomass with essential secondary macronutrients (Mg²^+^ and SO_4_²^-^). While future *in vivo* soil and greenhouse validations are required to confirm field-scale efficacy, this work establishes a quantitative proof-of-concept for strategically shifting intracellular carbon fluxes to produce functionally enhanced, sodium-free microalgal bio-fertilizer feedstocks. Ultimately, this bioprocess offers a sustainable, resource-efficient pathway to support closed-loop agricultural nutrient cycling and the circular bioeconomy.

## Data Availability

The datasets presented in this study can be found in online repositories. The names of the repository/repositories and accession number(s) can be found in the article/Supplementary Material.
